# Pediatric blood transfusion practices at a regional referral hospital in Kenya

**DOI:** 10.1111/trf.13774

**Published:** 2016-09-09

**Authors:** Helen M. Nabwera, Greg Fegan, Jay Shavadia, Douglas Denje, Kishor Mandaliya, Imelda Bates, Kathryn Maitland, Oliver W. Hassall

**Affiliations:** ^1^ Centre for Geographic Medicine Research (Coast)Kenya Medical Research Institute/Wellcome Trust Research ProgrammeKilifiKenya; ^2^ Liverpool School of Tropical MedicineLiverpoolUK; ^3^ Centre for Clinical Vaccinology & Tropical MedicineUniversity of OxfordOxfordUK; ^4^ Swansea Trials UnitSwansea University Medical SchoolSwanseaUK; ^5^ The Aga Khan University HospitalNairobiKenya; ^6^ Coast Provincial General HospitalMombasaKenya; ^7^ Regional Blood Transfusion CentreMombasaKenya; ^8^ Department of MedicineImperial CollegeLondonUK; ^9^ Department of Primary Care Health SciencesUniversity of OxfordOxfordUK

## Abstract

**BACKGROUND:**

Severe anemia in children is a major public health problem in sub‐Saharan Africa. In this study we describe clinical and operational aspects of blood transfusion in children admitted to Coast Provincial General Hospital, Kenya.

**STUDY DESIGN AND METHODS:**

This was an observational study where over a 2‐year period, demographic and laboratory data were collected on all children for whom the hospital blood bank received a transfusion request. Clinical data were obtained by retrospective review of case notes over the first year.

**RESULTS:**

There were 2789 requests for blood for children (median age, 1.8 years; interquartile range [IQR], 0.6‐6.6 years); 70% (1950) of the samples were crossmatched with 85% (1663/1950) issued. Ninety percent (1505/1663) were presumed transfused. Median time from laboratory receipt of request to collection of blood was 3.6 hours (IQR, 1.4‐12.8 hr). Case notes of 590 children were reviewed and median pretransfusion hemoglobin level was 6.0 g/dL (IQR, 4.2‐9.1 g/dL). Ninety‐four percent (186) were transfused “appropriately” while 52% (120) were transfused “inappropriately.” There was significant disagreement between the clinical and laboratory diagnosis of severe anemia (exact McNemar's test; p < 0.0001). Antimalarials were prescribed for 65% (259) of children who received blood transfusions but only 41% (106) of these had a positive blood film.

**CONCLUSION:**

In this setting, clinicians often order blood based on the clinical impression of “severe anemia.” This has implications for laboratory workload and the blood supply itself. However, the majority of children with severe anemia were appropriately transfused. The use of antimalarials with blood transfusions irrespective of blood film results is common practice.

**ABBREVIATIONS**
CPGH: Coast Provincial General Hospital
KNBTS: Kenya National Blood Transfusion Services
WAZ: weight‐for‐age z‐scores

Severe anemia resulting in significant morbidity and mortality is common in children in sub‐Saharan Africa and urgent blood transfusion is a lifesaving intervention.^1^, ^2^, ^3^, ^4^ Blood shortages are common in low‐ and middle‐income countries and delays in the acquisition and administration of blood have contributed significantly to in‐hospital mortality of children with severe anemia.^5^

International clinical transfusion guidelines for settings with limited resources are restrictive and reflect historical inadequacies in the blood supply. Since 2005, the World Health Organization (WHO) recommendation has been to reserve transfusion for those children with profound anemia (hemoglobin [Hb] ≤ 4 g/dL; Hb ≤ 10 g/dL in neonates) and in those where anemia is less severe (Hb 4‐6 g/dL) only when signs of critical illness are present. Whole blood transfusion of 20 mL/kg is recommended (10 mL/kg red blood cells [RBCs] preferred when available for children with heart failure and severe acute malnutrition) and for children with severe acute malnutrition 10 mL/kg whole blood and furosemide (1 mg/kg intravenously) at the start of the transfusion.^6^ In contrast, in high‐income settings transfusion guidelines for hemoglobin (Hb) thresholds in children and neonates are between 7 and 12 g/dL depending on the clinical context and RBCs are routinely available and used.^7^, ^8^

To prevent the transmission of malaria by blood transfusion in endemic countries, international guidelines recommend donor selection and deferral strategies or laboratory screening for malaria of all blood donations.^9^ In practice, these two strategies are rarely implemented and a third approach is also recommended—the administration of malarial chemoprophylaxis after transfusion, especially to the most vulnerable, that is, pregnant women and children.^10^ The Kenya National Blood Transfusion Services (KNBTS) that was established in 2001 has adopted this policy, which has not been revised in their latest guidance from 2004.^11^ In addition, blood is routinely screened for human immunodeficiency virus, for hepatitis B and C, and for syphilis but not for bacterial infection.^11^, ^12^

In the past decade the establishment of national blood transfusion services with impetus from the WHO “Goal of African Regional Blood Safety by 2012” and the mobilization of voluntary, nonremunerated blood donors have significantly improved the supply and safety of blood in many countries in sub‐Saharan Africa, including Kenya.^12^, ^13^ Nevertheless, blood remains a limited and potentially hazardous resource and one that should be given in a timely and safe manner to only those that need it.^14^, ^15^ There are few data relating to the clinical and operational aspects of pediatric blood transfusion in Kenya since the introduction of the national blood service in 2001.^3^, ^4^, ^16^, ^17^ The aim of this observational study was to describe clinical and laboratory practice and identify potential areas for intervention to improve the efficacy and safety of blood transfusion for children admitted to the second largest public referral hospital in Kenya.

## MATERIALS AND METHODS

The study was conducted at Coast Provincial General Hospital (CPGH) in Mombasa. The Mombasa Regional Blood Transfusion Centre, which is one of the six regional centers of the KNBTS, is adjacent to CPGH and has provided the hospital with whole blood from nondirected donors since 2002. Blood supplied by the Mombasa Regional Blood Transfusion Centre has supplanted the hospital's replacement donor system and blood shortages are less frequent.

All requests to the hospital laboratory for blood for transfusion for children aged less than 14 years were identified prospectively over a 24‐month period. Data relating to transfusion requests, crossmatches, and issues were extracted from existing handwritten ledgers in the CPGH blood bank. There was no “group and save” option on the transfusion request form and most requests were marked “urgent.” There were no pediatric blood packs and blood volumes of less than 1 unit (450 mL) issued by the blood bank were drawn from standard blood packs into a dry bag.^14^ The study was undertaken from May 2005 to April 2007 and for the first 12 months, clinical case notes were sought from the hospital records department for all children for whom blood had been requested after they had been discharged or died. The hospital records department archived the notes of children who had died separately from those discharged alive. Data extraction relating to clinical case management including the clinician's indication for requesting blood, the volumes of blood they requested and prescribed, as well as whether or not the child received the blood transfusion was undertaken by two clinicians (OWH, JS) using a predefined template.

Data were entered onto an electronic database (Epidata)^18^ and exported to statistical software (STATA 11.2, StataCorp) for analysis. Where the laboratory pretransfusion Hb results were recorded in the notes they were used to classify children as having severe anemia or not according to the WHO criteria.^5^ Weight‐for‐age z‐scores (WAZ) were calculated using WHO 2006 child growth standards.^19^

We calculated a recognized measure of the efficiency of blood ordering and utilization—the “crossmatch‐to‐transfusion ratio” defined as the number of blood units crossmatched divided by the number of units transfused.^20^ We also calculated the “request‐to‐issue ratio” as the number of blood units requested divided by the number of units transfused to assess for mismatch between clinician's requests for blood and blood bank's issuing of blood.

Fisher's exact and chi‐square tests were used to compare categorical variables; continuous variables were summarized by means with 95% confidence intervals (CIs) and differences tested for significance using the t test. Nonnormally distributed data were summarized using medians and interquartile ranges (IQRs), and differences were tested for significance using the Wilcoxon‐Mann‐Whitney U test. We used McNemar's test to assess agreement between clinical and laboratory diagnosis of severe anemia. We estimated the positive percent agreement and the negative percent agreement using the laboratory diagnosis of severe anemia as a reference. A p value of less than 0.05 was considered significant.

## RESULTS

From May 2005 to April 2007, there were 17,558 pediatric admissions to CPGH and 2789 pediatric blood transfusion requests. Blood was crossmatched for 1950 (70%) of the requests and in 1505 cases crossmatched blood was issued from the blood bank and not returned. Therefore, 77% (1505/1950) of crossmatched blood and 54% (1505/2789) of requests for transfusion were presumed transfused (crossmatch‐to‐transfusion ratio, 1.3; request‐to‐issue ratio, 1.9). In 156 cases, crossmatched blood was collected from the blood bank but returned unused. Of the 2514 transfusion requests made to the blood bank where the blood volume for whole blood was recorded, 50% were for less than 1 unit, 38% were for 1 unit, and 12% were for greater than 1 unit. Of the 1845 crossmatches by the blood bank where the blood volume was recorded, 45% were for the issue of either less than 1 unit (44%) or greater than 1 unit (1%; 62% vs. 45%; chi‐square; p < 0.0001). Fifty‐five percent of the blood issued by the blood bank was for 1 unit whereas only 38% of requests were for 1 unit (55% vs. 38%; Fisher's exact test; p < 0.0001). The majority of requests (50%) made were for less than 1 unit of blood (Table 1).

**Table 1:** Pediatric transfusion requests and crossmatches over a 2‐year period at CPGH, Kenya

Characteristics	Requested	Crossmatched
Number (%)	2789 (100)	1950 (100)
Age (years), mean (IQR)	1.75 (0.6‐6.0)	‐
Female, n (%)	1188 (43)	‐
Blood volumes*	‐	‐
Observations, n (%)	2514 (90)	1847 (95)
Median, mL (IQR)	400 (200‐450)	450 (200‐450)
Range, mL	10‐1800	2‐900
Mean, mL (±SD)	370 (±238)	338 (±145)
<1 unit, n (%)	1267 (50)†	812 (44)†
1 unit, n (%)	948 (38)†	1022 (55)†
>1 unit, n (%)	299 (12)†	11 (1)†

*Volumes of 1 unit and greater are multiples of 1 unit (450 mL).

†Fisher's exact test for difference between volumes requested and prescribed, p < 0.0001.

Despite repeated attempts, it was only possible to retrieve case notes for 590 (45%) of the 1322 children for whom blood was requested over the first 12 months of the study; 67% (396/590) of these children received a blood transfusion. A clinical diagnosis of severe anemia by the attending clinician was recorded in 48% (281/590) of the case notes, and 88% (248/281) of these were transfused. Of the 492 children who had a pretransfusion Hb recorded in their notes, only 66% (327/492) had severe anemia using the WHO definition and 88% (287/327) of these were transfused (Table 2). Twenty‐four percent (141/590) of children had neither a clinical nor a laboratory diagnosis of severe anemia and 27% (38/141) of these were transfused. The median pretransfusion Hb concentration was 10.2 (IQR, 6.5‐13.2) g/dL in children who were 3 months old or younger and 5.6 (IQR, 4.0‐8.4) g/dL in those over 3 months of age.

**Table 2:** Summary characteristics of all children whose case notes were reviewed

Characteristics	N = 590	Transfused (n = 396)	Furosemide (n = 349)	Antimalarial (n = 294)
Age (years), median (IQR)	1.38 (0.5‐4.0)	‐	‐	‐
Weight (kg), median (IQR)	8.0 (5.5‐12.0)	‐	‐	‐
Pretransfusion Hb recorded, n (%)	492 (83)	‐	‐	‐
Age < 3 months, n (%)	84 (14)	39 (46)	38 (45)	10 (12)
Severe anemia (clinical), n (%)	281 (48*)	248 (88)	231 (82)	216 (77)
Severe anemia (laboratory), n (%)	327 (66†)	287 (88)	246 (75)	221 (68)
Positive blood film, n (%)	137 (23‡)	116 (85)	106 (77)	118 (86)
WAZ score < −3§	112 (29||)	68 (54)	63 (56)	51 (46)
Discharged alive	458 (78)	315 (69)	260 (57)	236 (52)

*Denominator = 584.

†Denominator = 496.

‡Denominator = 332.

§Computed using WHO growth standards 2006.

ǁDenominator = 380.

We excluded 84 children aged less than 3 months of age and 23 children with no recorded data on “clinical severe anemia” from subsequent analyses as the WHO transfusion guidelines did not apply to this younger age group. The clinical impression of “severe anemia” was used as a proxy for the presence of one or more signs of critical illness (clinically detectable dehydration, shock, impaired consciousness, heart failure, deep labored breathing, very high malaria parasitemia). This clinical feature was recorded consistently and therefore allowed us to estimate adherence to WHO guidelines for transfusion (Table 3). Of the 197 children with either laboratory Hb level of not more than 4 or 4 to 6 g/dL and a clinical impression of severe anemia, 186 (94%) were transfused appropriately. Of the 232 children with either a Hb level of 4 to 6 g/dL and no severe anemia or a Hb level of more than 6 g/dL, 120 (52%) were transfused inappropriately (Table 3).

**Table 3:** Appropriate use of blood

Hb (g/dL)	Number (%)	Transfused (%)	Clinical severe anemia? (%)	Transfused (%)	Not transfused	Request to issue time (hr), median (IQR)
≤4	109 (26)	105 (96)	Yes 98 (89)	96 (98)	2	5.4 (2.3‐12.1)
			No 11	9	2	3.6 (1.7‐13.5)
4‐6	122 (29)	111 (91)	Yes 88 (72)	81 (92)	7	4.5 (1.6‐12.5)
			No 34	30	4	11.2 (4.6‐22.6)
> 6	196 (46)	90 (64)	Yes 44 (22)	34 (77)	10	5.8 (1.5‐18.2)
			No 152	56	96	6.2 (2.4‐21.3)
Total	424	342	‐	‐	121	‐

Time intervals from the receipt of a transfusion request to the issue of crossmatched blood were significantly shorter in those children with a clinical diagnosis of severe anemia compared to those without (Wilcoxon rank‐sum test; p = 0.01), but this was not the case in those children with a laboratory diagnosis of severe anemia (Wilcoxon rank‐sum test; p = 0.44). There was strong evidence of disagreement between the clinical and laboratory diagnosis of severe anemia (exact McNemar's test; p < 0.0001). Using the laboratory diagnosis as the reference, the positive percent agreement and negative percent agreement for clinical diagnosis were 69.2% (95% CI, 63.8%‐74.1%) and 83.2% (95% CI, 75.7%‐88.7%), respectively (Table 4).

**Table 4:** Comparison between laboratory and clinical diagnosis of severe anemia

Clinical diagnosis (n = 427)	Laboratory diagnosis (n = 427)		p value*	Overall proportion of agreement (95% CI)†	Positive percent agreement (95% CI)†	Negative percent agreement (95% CI)†
	Yes	No				
Yes	209	21	<0.0001	73.3 (68.9‐77.3)	69.2 (63.8‐74.1)	83.2 (75.7‐88.7)
No	93	104				

*Exact McNemar's test.

†Wilson 95% CIs. Positive percent agreement and negative percent agreement were estimated using laboratory diagnosis as the reference.

The median volume of whole blood prescribed was 200 mL (IQR, 150‐336 mL), and the median volume prescribed by weight was 22.1 mL/kg (IQR, 20‐30 mL/kg; Table 4). Eighty‐one percent (334/410) of blood volumes prescribed by clinicians were for less than 1 unit, and 15% (60/410) were for 1 unit (450 mL). In 33% (118/354) of cases, clinicians requested a larger volume of whole blood than that which they had prescribed. Of blood transfusion requests made by clinicians and received in the blood bank, 66% (293/442) were for less than 1 unit and 27% (119/442) for 1 unit. Of the 367 crossmatches by the blood bank, 47% (172) were for the issue of 1 unit (Table 5).

**Table 5:** Comparison of volumes of blood prescribed, requested, and crossmatched in children in whom case notes were reviewed*

Characteristics	Requested	Prescribed	Crossmatched
Number (%)	442 (75)	410 (69)	367 (62)
Volume (mL), median (IQR)	250 (300)	200 (186)	340 (250)
Range (mL)	10‐900	14‐900	15‐600
Mean (±SD)	300 (±207)	252 (±155)	312 (±146)
<1 unit (%)	293 (66)	334 (81)	193 (53)
1 unit (%)	119 (27)	60 (15)	172 (47)
>1 unit (%)	30 (7)	16 (4)	2 (<1)
Blood volumes (mL/kg)			
Number (%)	442 (75)	378 (64)	367 (62)
Volume, median (IQR)	25 (20‐39.5)	22.1(20‐30)	28.6 (20.5‐45)
Range	2.5‐360	7.1‐66.7	3.3‐409.1

*Case notes of 590 children were reviewed.

Of the 396 children whose case notes were reviewed and who were transfused, 259 (65%) were prescribed an antimalarial and of those who were prescribed antimalarials, 106 (41%) had a positive blood film, 97 (38%) had a negative blood film, and 55 (21%) had no result of a blood film recorded. Furosemide was prescribed for 318 (81%) of the children transfused and for 27 (7%) of those who were not. Of the 112 children with WAZ of less than −3 (indicating that they were severely underweight), 71 were transfused. Of these, one (0.5%) received 10 mL/kg or less of whole blood (median, 25.8 mL/kg; IQR, 20‐32 mL/kg), and 63 (77%) were prescribed furosemide (Table 2).

## DISCUSSION

To our knowledge this is the first detailed description of the operational aspects of clinical and laboratory blood transfusion practice in children (n = 2789) admitted to a Kenyan hospital since the KNBTS was established in 2001. Blood transfusion is an integral part of acute pediatrics where children's lives are saved if they receive appropriate blood transfusions in a timely manner.^8^ However, it can also be a very costly intervention if done inappropriately.^15^, ^17^ A clear understanding of these operational aspects is therefore important in improving the efficiency and delivery of this vital service.

Ninety‐six percent of children with laboratory‐confirmed severe anemia were transfused appropriately but there was significant overtransfusion of children who did not fulfill the criteria for transfusion (52%), that is, 120 inappropriate transfusions in children over a period of a year. The data indicate that the clinicians were likely to be deciding to transfuse on the basis of their clinical judgment without laboratory confirmation of the level of anemia. This may have been because they did not trust the Hb results, the results did not come back quickly enough, or that they did not send the Hb tests and/or wait for the result.^1^, ^21^ The use of pallor to screen for severe anemia has good specificity (88%‐95%) but poor sensitivity (27%‐50%) and results in patients who are not severely anemic receiving unnecessary transfusions.^22^, ^23^ There is therefore a need to have quality assurance mechanisms to improve the accuracy and rapidity of Hb results available through the laboratory to clinicians. Clinicians would then be able to demonstrate that their blood request is based on a laboratory Hb level. For urgent requests this check of Hb could be done by the blood bank staff and senior staff should query clinicians about the need for transfusion if the Hb is not low enough. This would dissuade clinicians from requesting for blood based on a clinical diagnosis alone. Overordering of blood wastes a scarce resource as well as the blood bank staff time, which contributes to delays in crossmatching of blood. However, we recognize that the provision of a reliable rapid Hb result may not necessarily dissuade clinicians from relying on their clinical diagnosis of severe anemia, as has been shown with the use of malaria rapid diagnostic tests and prescribing of antimalarials for patients with negative results.^24^ More research is needed to explore why and how behavior might be changed.

Clinicians generally prescribed blood according to the WHO recommendation of 20 mL/kg,^5^ but 33% of requests were for larger volumes of blood than were actually prescribed on the patient charts. This may suggest a lack of confidence between clinical and laboratory services—whereby clinicians' expectation that the laboratory might ration the transfusion volume to be issued leads them to increase the requested volumes in the hope of receiving an appropriate amount. Alternatively, this discrepancy may be due to the fact that blood request procedures may not have been updated in line with the WHO guidelines and therefore blood is ordered in units and not in milliliters, therefore resulting in larger volumes being requested. Training of clinicians in the indications for blood transfusions in children and improved communication and working relations between clinicians and blood bank staff through a hospital transfusion committee might be a possible solution to this.^25^

The discrepancy between the volumes of blood requested by clinicians and those crossmatched by blood bank is likely to be related to the lack of pediatric packs. The blood bank is probably, and rightly, wary of entering packs too often to withdraw aliquots of blood because of the risk of introducing infection. Although there may be additional expense associated with purchasing small‐volume pediatric packs, this may be offset against better matching of volume against need and therefore result in less waste. This would need to be explored in a comparative study. There are also significant risks of overtransfusion associated with this practice on wards that are often poorly staffed with a lack of burettes to aid with monitoring the volumes of blood being transfused to the children. However, there is some evidence that the current WHO blood transfusion recommendations of 20 mL/kg whole blood or 10 mL/kg RBCs may undertreat a significant proportion of anemic children but further research is required to build on this evidence.^26^

A median request‐to‐issue time of between 3.6 and 5.4 hours is unacceptably prolonged for urgent requests for blood. This may have been due to shortages of blood or the perception of unnecessary requests for blood, leading the blood bank to wait for additional indications that the blood was really required such as a phone call or ward staff attending the blood bank in person to chase the request. This affects patient care as delays in transfusing these sick children can often lead to adverse outcomes.^26^ Potential quality improvement interventions would include introducing a group and save option for clinicians supported by an agreement by the blood bank to be able to provide blood within 4 hours if it was requested and clinicians would need to understand that the sample would be unusable and therefore destroyed after a specific time had elapsed. In addition, a formal “urgent” request can be introduced, whereby the blood bank would undertake to provide blood within 2 hours. Shortages of blood became less frequent with the establishment of a regional blood transfusion center on the same campus as the hospital.

Most of the children who were transfused received antimalarial therapy irrespective of blood film results. As donated blood was not routinely screened for malaria in Kenya at the time of the study, clinicians would have prescribed antimalarials in children receiving blood transfusions due to a perceived risk of transfusion‐induced malaria as recommended in the Kenyan blood transfusion guidelines.^25^ This practice although pragmatic, does not eliminate the risk of transfusion‐induced malaria.^27^, ^28^, ^29^ This is costly particularly in areas where there is chloroquine resistance and artemesinins have to be used.^30^ It also contradicts WHO guidance about only using antimalarials for proven malaria.^5^ However, there is no suitable screening test for this in the context of blood donor screening in endemic areas and eliminating malaria‐positive donors is likely to have a significant effect of reducing the blood supply and therefore increasing mortality.^31^ Pathogen inactivation technologies are a potential solution for the future.^32^ More research is needed about how to change the behavior of clinicians to comply with these changes (e.g., education, better supervision of transfusion practice, blood bank being able to query requests), how to establish and maintain transfusion committees, and the cost‐effectiveness of introducing specialist transfusion practitioners.

This study has a number of limitations. As we were not able to access the clinical records of all the children who had had a request for blood and there was separate archiving of records of those children that died, we were not able to estimate the overall survival or mortality outcomes in relation to the timing and receipt of blood transfusions. In addition, the recording of clinical data in the records was often incomplete resulting in missing data, which we have highlighted in our results. These data are also 8 to 10 years old; however, they are still relevant to the current operational aspects of pediatric blood transfusions in hospital in Kenya where blood remains scarce, as the demand is often greater than the supply with a decrease in blood donors.^11^ In addition, the KNBTS guidelines have not been updated since 2004 and therefore transfusion‐transmitted malaria remains a risk as screening of blood for malaria parasites is still not national policy and the recommendation remains to give antimalarial drugs to recipients of blood shortly after receiving the transfusion.^11^ In addition, we have been able to highlight important areas for intervention that could significantly improve the processes for pediatric blood transfusions at the clinical–blood bank interface in this hospital and other hospitals facing similar challenges in the region.

## CONCLUSION AND RECOMMENDATIONS

Our data suggest that in a setting with a high demand for pediatric transfusion but historically poor supply, clinicians tend to overorder blood in terms of both the number of requests and the volumes requested. This has implications for laboratory workload and the blood supply itself. This study provides information on areas that need to be strengthened within the system for pediatric blood transfusions in this large referral hospital in Kenya and recommendations that can help improve the efficacy and safety of blood transfusions for children in hospitals in Kenya and the region. This will ultimately improve the survival of very sick children who present to hospitals and require this lifesaving intervention.

## CONFLICT OF INTEREST

The authors have disclosed no conflicts of interest.

## Supporting information

Additional Supporting Information may be found in the online version of this article at the publisher's website:

**Table S1**. Clinical data extracted from case note review
