# Supplementary material for: Pediatric blood transfusion practices at a regional referral hospital in Kenya
Source: Transfusion. 2016 Sep 9;56(11):2732–8. doi: 10.1111/trf.13774 (PMC5111588; doi:10.1111/trf.13774)
Supplement: Supplementary file 1 — Table S1. Clinical data extracted from case note review [file TRF-56-2732-s001.docx]

**Paediatric blood transfusion practices at a regional referral hospital in Kenya**

Nabwera H M*^1^, Fegan G ^1, 2,3^, Shavadia J^4^, Denje D^5^, Mandaliya K ^5, 6^, Bates I ^7^, Maitland K ^1,8^ , Hassall O W^1, 6, 9^

1 Centre for Geographic Medicine Research (Coast), Kenya Medical Research Institute/ Wellcome Trust Research Programme, Kilifi 80108, Kenya

2 Centre for Clinical Vaccinology & Tropical Medicine, University of Oxford, Oxford OX3 7LJ, UK

3 Swansea Trials Unit, Swansea University Medical School, Swansea, SA2 8PP

4 The Aga Khan University Hospital, Nairobi, Kenya

5 Coast Provincial General Hospital, Mombasa 80100, Kenya

6 Regional Blood Transfusion Centre, Mombasa 80100, Kenya

7 Liverpool School of Tropical Medicine, Liverpool L1 5QA, UK

8 Department of Medicine, Imperial College, London, SW7 2AZ, UK

9 Department of Primary Care Health Sciences, University of Oxford, Oxford OX1 2ET, UK

Supplementary material

1. Supplementary table (1)

**Table S1: Clinical data extracted from case note review**

| Demographic | Anthropometry | Laboratory results | Details of blood transfusion |
| --- | --- | --- | --- |
| Name  Age (years, months, days)  Sex (M/F)  Ward  In patient number  Casualty number  Outpatient number  Date of admission  Time of admission  Date of discharge/death  Time of discharge or death | Weight (kg) | Blood slide  Admission Hb (g/dL) | Blood requested (Y/N)  On admission? (Y/N)  Date blood requested  Time blood requested  Volume of blood requested (ml)  Blood received (Y/N/X)  Date blood received  Time blood received  Blood transfusion completed (Y/N/X)  Frusemide received (Y/N)  Post transfusion Hb (g/dL)  Anaemia aetiology |
